# Identifying Adolescent Metabolic Syndrome Using Body Mass Index and Waist Circumference

**Published:** 2008-09-15

**Authors:** Sarah M Camhi, JoAnn Kuo, Deborah R Young

**Affiliations:** University of Maryland, College Park School of Public Health, Department of Kinesiology; University of Maryland, College Park School of Public Health, Department of Kinesiology, College Park, Maryland; University of Maryland, College Park School of Public Health, Department of Kinesiology, College Park, Maryland

## Abstract

**Introduction:**

Metabolic syndrome is increasing among adolescents. We examined the utility of body mass index (BMI) and waist circumference to identify metabolic syndrome in adolescent girls.

**Methods:**

We conducted a cross-sectional analysis of 185 predominantly African American girls who were a median age of 14 years. Participants were designated as having metabolic syndrome if they met criteria for 3 of 5 variables: 1) high blood pressure, 2) low high-density lipoprotein cholesterol level, 3) high fasting blood glucose level, 4) high waist circumference, and 5) high triglyceride level. We predicted the likelihood of the presence of metabolic syndrome by using previously established cutpoints of BMI and waist circumference. We used stepwise regression analysis to determine whether anthropometric measurements significantly predicted metabolic syndrome.

**Results:**

Of total participants, 18% met the criteria for metabolic syndrome. BMI for 118 (64%) participants was above the cutpoint. Of these participants, 25% met the criteria for metabolic syndrome, whereas only 4% of participants with a BMI below the cutpoint met the criteria for metabolic syndrome (*P* <.001). Girls with a BMI above the cutpoint were more likely than girls with a BMI below the cutpoint to have metabolic syndrome (*P =* .002). The waist circumference for 104 (56%) participants was above the cutpoint. Of these participants, 28% met the criteria for metabolic syndrome, whereas only 1% of participants with a waist circumference below the cutpoint met the criteria for metabolic syndrome (*P* <.001). Girls with a waist circumference above the cutpoint were more likely than girls with a waist circumference below the cutpoint to have metabolic syndrome (*P* = .002). Stepwise regression showed that only waist circumference significantly predicted metabolic syndrome.

**Conclusion:**

Both anthropometric measures were useful screening tools to identify metabolic syndrome. Waist circumference was a better predictor of metabolic syndrome than was BMI in our study sample of predominantly African American female adolescents living in an urban area.

## Introduction

Metabolic syndrome is defined as clustering of several cardiovascular and metabolic risk factors. Clinical diagnosis is made on the basis of the presence of 3 of 5 conditions: 1) high triglyceride level, 2) low high-density lipoprotein (HDL) cholesterol level, 3) high fasting blood glucose level, 4) presence of central obesity (ie, high waist circumference), and 5) high blood pressure (1). The metabolic syndrome classification is of interest in epidemiologic studies because of its ability to predict the development of cardiovascular disease (CVD) and type 2 diabetes in adults (2).

Metabolic syndrome and its individual components are detectable during childhood, and both commonly persist throughout adolescence and adulthood (3-5). The trend for metabolic syndrome is increasing nationally. The most current data show that a 9.2% prevalence of metabolic syndrome from 1988 through 1994 increased to 12.7% from 1999 through 2000 (6). The larger the clustering of risk factors is during childhood or adulthood, the greater the extent of atherogenic damage and disease severity (7). Therefore, identifying metabolic syndrome during childhood is vital to curbing the development and progression of cardiovascular and metabolic disease during adulthood.

Early screening for metabolic syndrome can be expensive and time-consuming because it involves testing for multiple risk factors. Although the prevalence among adolescents is increasing, it is still low and routine laboratory screening is not needed. However, a simple and easy screening procedure that uses a single measurement can help determine whether further testing is needed. Katzmarzyk et al previously published sex-, age-, and race/ethnicity-specific body mass index (BMI) and waist circumference cutpoints to identify adolescents at risk for CVD risk factor clustering (8). Because Katzmarzyk et al did not determine whether these cutpoints identified metabolic syndrome, we attempted to do so in a sample of predominantly African American adolescent girls who attended high school and who lived in an urban area.

## Methods

We analyzed baseline data collected for Project Heart, an intervention trial in a Baltimore, Maryland, high school (9). The intervention was designed to increase physical activity levels and fitness among high school girls.

Project Heart participants were recruited during the fall of 2000, 2001, and 2002 from ninth-grade students at a public magnet high school with an all-female student body. The school has a college-preparatory curriculum and draws students from most neighborhoods in the city. Participants were recruited through presentations made to students and parents during summer freshman-orientation classes; presentations made to ninth-grade girls enrolled in eligible physical education classes; and letters sent from the principal to families, along with brochures explaining study details and appropriate forms for participation. Eligibility inclusion criteria were willingness and ability to participate in all aspects of the intervention, being enrolled in ninth grade at the high school, being enrolled in 2 consecutive semesters of physical education, and parental/guardian consent. Exclusion criteria were having a medical condition that excused a student from meeting the Maryland Physical Education Content Standards (10), being pregnant or breastfeeding, planning to leave the area before follow-up, participating currently in another clinical trial that conflicted with the study, or having another household member participating in Project Heart. A total of 221 ninth-grade girls were recruited into the trial. Blood samples were available from 185 participants, and these participants were included in this study. Study protocol was approved by the institutional review boards of Johns Hopkins University and the University of Maryland.

Baseline measurements were collected during the first 4 weeks of the fall semester before school, after school, during physical education class, or during lunch. Ethnicity was self-reported. Trained technicians collected all measurements. Participants removed their shoes and outer clothing before weight and height measurements were taken. Technicians took 3 waist circumference measurements— each rounded to the nearest 0.1 cm — from a horizontal plane 2.5 cm above the umbilicus and averaged them. Weight was measured by using a calibrated physician scale and was rounded to the nearest 0.1 kg. Height was measured using a standard wall stadiometer and was rounded to the nearest 0.1 cm. BMI was calculated from weight (kg) divided by the squared height (m^2^).

Resting blood pressure was obtained from the nondominant arm by using an automated blood pressure monitor (DINAMAP, GE Medical Systems Information Technologies, Inc, Milwaukee, Wisconsin) that is a reliable and valid instrument, particularly when 2 or more measurements are averaged (11). Participants rested in a seated position for 5 minutes before measurements were taken. Three blood pressure measurements were taken, with a 1-minute interval between measurements, and averaged. Three 30-second peripheral pulse rates were also taken and averaged.

Participants' blood profiles were obtained after a 12-hour fast. Samples were sent to a commercial laboratory (Quest Diagnostics, Lyndhurst, New Jersey) for analysis. Total cholesterol, HDL cholesterol, low-density lipoprotein cholesterol, glucose, and triglyceride levels were measured by using standard laboratory procedures.

We used the definition of metabolic syndrome described by de Ferranti et al (12). Participants were defined as having metabolic syndrome if they met or exceeded the criteria for 3 or more of the following 5 variables: 1) triglycerides ≥1.1 mmol/L; 2) HDL cholesterol <1.3 mmol/L; 3) fasting blood glucose ≥6.1 mmol/L; 4) waist circumference (cm) >75th percentile for age and sex; and 5) systolic or diastolic blood pressure (mm Hg) >90th percentile for age, sex, and height.

BMI and waist circumference cutpoints established by Katzmarzyk et al (8) were applied to identify participants who had metabolic syndrome. Table 1 displays the age- and ethnicity-specific cutpoints for BMI and waist circumference.

### Statistical methods

We calculated means of the continuous variables and frequencies of the categorical variables and used the data to describe CVD risk factor characteristics of participants with and without metabolic syndrome. We used general linear modeling to assess age- and race/ethnicity-adjusted means and the differences between participants with and without metabolic syndrome.

We conducted *χ* ^2^ tests to determine whether the distributions of participants with and without metabolic syndrome varied from participants with BMI and waist circumference above and below the cutpoints (8). We performed logistic regression analysis to determine whether measurements exceeding the BMI and waist circumference cutpoints were predictive of metabolic syndrome. We performed stepwise logistic regression to determine whether BMI or waist circumference was a stronger predictor of metabolic syndrome. All logistic regression analyses were adjusted for age and ethnicity. Data were analyzed by using SAS version 9.1 (SAS Institute, Cary, North Carolina).

## Results

No demographic differences existed between participants who did and who did not provide blood samples. Characteristics of the participants are presented in Table 2. According to the international definition for childhood overweight and obesity (13), 51% of participants were normal weight, 21% were overweight, and 28% were obese. Most participants were African American, 12% were white, and 4% reported other race/ethnicity. This demographic composition was similar to the overall school population. Most participants were approximately aged 14 years. Approximately 18% of participants had metabolic syndrome.

Significant differences were found between girls with metabolic syndrome and girls without metabolic syndrome for triglyceride level, HDL cholesterol level, fasting blood glucose level, BMI, waist circumference, systolic blood pressure, and diastolic blood pressure (Table 3). The 3 variables most commonly associated with the participants with metabolic syndrome were high waist circumference (94%), low HDL cholesterol level (91%), and high diastolic blood pressure (82%) (Table 4).

Of all participants, 118 (64%) had a BMI that exceeded the cutpoint that indicates risk for metabolic syndrome (8). Of those participants, 25% met the criteria for metabolic syndrome. Of participants whose BMI fell below the cutpoint, 4% met the criteria for metabolic syndrome (*P* < .001) (Table 5). Participants with a BMI above the cutpoint were more likely than participants with a BMI below the cutpoint to have metabolic syndrome (adjusted odds ratio [AOR], 10.2; 95% confidence interval [CI], 2.30-45.46; *P* = .002).

Of total participants, 104 (56%) had a waist circumference that exceeded the cutpoint that indicates risk for metabolic syndrome. Of those participants, 28% met the criteria for metabolic syndrome. Of participants with a waist circumference that fell below the cutpoint, 1% met the criteria for metabolic syndrome (*P* < .001) (Table 5). Participants with a waist circumference above the cutpoint were more likely than participants with a waist circumference below the cutpoint to have metabolic syndrome (AOR, 24.3; 95% CI, 3.23-182.89; *P* = .002). Waist circumference significantly predicted metabolic syndrome (AOR, 25.6; 95% CI, 3.42-192.18; *P* = .002) (data not shown).

## Discussion

BMI and waist circumference cutpoints previously used to identify CVD risk factor clustering were successful in identifying metabolic syndrome in adolescent girls. Because waist circumference had a stronger predictive ability than did BMI in identifying girls with metabolic syndrome, it should be used to screen adolescent girls for metabolic syndrome.

Previous international studies of children and adolescents (14-19) and American studies of prepubescent children (20) have demonstrated that high waist circumference or high intra-abdominal adiposity is a better predictor of metabolic syndrome than is BMI. A few studies have reported the association between clustering of risk factors for metabolic syndrome and BMI or waist circumference in American adolescents (21,22), but none have examined metabolic syndrome in American adolescents. Our results add to the body of knowledge on American adolescents by confirming that the waist circumference cutpoint identified risk for metabolic syndrome in adolescents and that a waist circumference that exceeded the cutpoint was a stronger predictor of metabolic syndrome in adolescents than was a BMI that exceeded the cutpoint.

Waist circumference may be a better predictor than BMI in detecting metabolic syndrome because of its association with increased visceral adipose tissue. Increased visceral adipose tissue is associated with insulin resistance, glucose intolerance, and abnormal lipid profiles and is an independent predictor for the development of type 2 diabetes in adults (23). The metabolic activity of visceral fat can increase free fatty acid circulation, decrease insulin uptake by the liver, increase circulating insulin levels, and ultimately lead to glucose intolerance (24). Furthermore, insulin resistance impairs the breakdown of triglycerides, which in turn stimulates the production of other atherogenic lipoproteins and decreases HDL cholesterol levels (24).

Although visceral adipose tissue increases the chance that adults will develop metabolic syndrome (25), this relationship is more difficult to establish in adolescents. Because of hormonal and maturational differences between adolescents and adults, adolescents tend to have lower visceral fat deposits than do adults (26). However, visceral fat is acquired during maturation in proportion to the increase in general body fat, which may suggest that visceral fat accumulation is as relevant a risk factor in adolescence as it is in adulthood (27). Thus, waist circumference as a measure of central adiposity may be a more specific and sensitive tool than BMI in identifying multiple elevated risk factors for CVD, and specifically metabolic syndrome, in adolescents.

The Katzmarzyk cutpoints and the metabolic syndrome definition include a measure of waist circumference. However, the Katzmarzyk cutpoints and the definition for metabolic syndrome are different because they were developed on the basis of 2 different outcomes. The Katzmarzyk cutpoints were created to optimally predict where multiple elevated risk factors occur by using sensitivity and specificity curves for age-, sex-, and race/ethnicity-specific criteria (8). For example, in terms of risk factor clustering, a 13-year-old African American girl has a waist circumference cutpoint of 68.4 cm. If a participant's waist circumference is above the cutpoint, then the participant is more likely to have other elevated risk factors. In contrast, the waist circumference criterion used for the metabolic syndrome definition acts as a threshold to indicate the presence of a *single* risk factor (12). Using the de Ferranti et al definition of metabolic syndrome (12), the waist circumference criterion is identified as being above the 75th percentile determined by age and sex (28). Thus, for a 13-year-old African American girl, the waist circumference threshold is 78.8 cm. Meeting this criterion is insufficient to classify metabolic syndrome, as she has met only 1 of the 5 criteria for diagnosing metabolic syndrome. Therefore, the cutpoints from Katzmarzyk et al (8) predict the point at which cardiovascular clustering is more likely to occur, whereas the de Ferranti et al definition for meeting the waist circumference cutpoint (12) only identifies 1 of the 5 criteria for meeting the definition of metabolic syndrome.

Multiple definitions of metabolic syndrome make it difficult to directly compare population prevalence among studies. We found a prevalence of metabolic syndrome of 18% in a sample of predominantly African American, mostly overweight or obese adolescent girls. Cook et al found a 4% overall prevalence of adolescent metabolic syndrome and a 28% prevalence among overweight adolescents (29). De Ferranti et al, using the definition of metabolic syndrome that we used for our study, found an overall prevalence of adolescent metabolic syndrome of approximately 12% (12). However, 30.5% of overweight adolescents were identified as having metabolic syndrome (7). Thus, the prevalence of metabolic syndrome in our sample is comparable, although slightly lower, to previous work done among overweight adolescents.

The main goal of our research was to determine whether previously defined age- and race/ethnicity-specific cutpoints of BMI and waist circumference had predictive value for identifying metabolic syndrome. Therefore, we did not conduct a sensitivity or specificity analysis to evaluate optimal cutpoints. Future work should consider the use of the Katzmarzyk et al cutpoints relative to those determined from a sensitivity analysis in adolescent populations (8). If the Katzmarzyk cutpoints are reasonably approximated, they may be useful tools for clinical screening for metabolic syndrome (8).

Although BMI is easy to measure, the measurement requires using a calibrated scale, obtaining the height and weight of a participant, and performing a calculation. Removal of shoes and heavy clothing is required by the participant. In contrast, waist circumference is easier to obtain because it is a single measurement, requiring only an inexpensive tape measure. Obtaining the measurement poses minimal participant burden because clothing needs to be removed only from the abdominal area. Waist circumference is easy to measure, fast, cheap, and highly reproducible (30). Therefore, the Katzmarzyk waist circumference cutpoints for adolescent girls (8) are preferred to the BMI cutpoints, both in their predictive ability and practical application, for screening for metabolic syndrome.

This sample included a high percentage of African American girls, which may limit the generalizability to other adolescents. However, a high percentage of young African American girls are overweight (31), have reduced insulin sensitivity (32), have at least 1 risk factor for metabolic syndrome (33), and have type 2 diabetes (34). Therefore, we could apply the Katzmarzyk cutpoints and established statistical associations in this at-risk population, which provided sufficient evidence of metabolic syndrome (8). The narrow age range (13-15 years) of participants also limited the generalizability of our results.

Both BMI and waist circumference cutpoints accurately detected metabolic syndrome in a predominantly African American sample of female adolescents. Waist circumference was a better predictor of metabolic syndrome than was BMI. Therefore, health care practitioners should routinely measure waist circumference when screening adolescents.

## Figures and Tables

**Table 1:** Body Mass Index and Waist Circumference Cutpoints^a^ Used to Identify Metabolic Syndrome, by Participant Age and Ethnicity, Baltimore, Maryland, 2000-2002

**Characteristic**		Variables and Cutpoints	

Race/Ethnicity	Age, y	Body Mass Index (kg/m^2^)	Waist Circumference (cm)
White	13	≥21.1	≥69.7
	14	≥21.6	≥70.9
	15	≥21.9	≥71.3
African American	13	≥20.5	≥68.4
	14	≥21.3	≥70.0
	15	≥22.1	≥71.5

a Cutpoints established by Katzmarzyk et al (8).

**Table 2:** Characteristics of Participants (N = 185) Assessed for Metabolic Syndrome, Baltimore, Maryland, 2000-2002

**Characteristic**	Value
**Mean age, y (SD)**	13.8 (13.35-14.25)
**Race/ethnicity, %**	
African American	84
White	12
Other	4
**Mother's education,^a^ %**	
≤High school	17
>High school	83
**BMI grouping,^b^ %**	
Normal weight	51
Overweight	21
Obese	28
**Mean height, cm (SD)**	162.4 (155.43-169.38)
**Mean weight, kg (SD)**	67.1 (46.42-87.78)
**Mean BMI, kg/m^2^ (SD)**	25.3 (18.40-32.20)

Abbreviations: SD, standard deviation; BMI, body mass index.

a Mother’s education is used as a proxy measure for socioeconomic status.

b According to the international definition of childhood overweight and obesity (13).

**Table 3:** Cardiovascular Disease Risk Factors Associated With Metabolic Syndrome Among Adolescent Girls, Baltimore, Maryland, 2000-2002

**Cardiovascular Disease Risk Factors**	Total Sample (N = 185), Mean (SD)	Girls Without MS (n = 151), Mean^a^	Girls With MS (n = 34), Mean^a^	*P* value^b^
Triglyceride level, mmol/L	0.7 (0.36-1.04)	0.7	1.1	<.001
HDL cholesterol level, mmol/L	1.4 (1.12-1.68)	1.4	1.1	<.001
Fasting blood glucose level, mmol/L	4.6 (3.97-5.23)	4.5	4.8	.03
Body mass index, kg/m^2^	25.3 (18.40-32.20)	22.2	31.5	<.001
Waist circumference, cm	78.1 (63.10-93.10)	71.8	94.1	<.001
Systolic blood pressure, mm Hg	109.9 (98.77-121.03)	106.3	120.6	<.001
Diastolic blood pressure, mm Hg	75.6 (68.37-82.83)	73.3	82.1	<.001

Abbreviations: SD, standard deviation; MS, metabolic syndrome; HDL cholesterol, high-density lipoprotein cholesterol.

a Mean values adjusted for age and ethnicity.

b *P* values calculated by using general linear modeling and adjusted for age and ethnicity. *P* values determine statistical significance between girls with and without MS.

**Table 4:** Percentage of Participants (N = 185) With Cardiovascular Disease Risk Factor Values Above the Cutpoint for Defining Metabolic Syndrome,^a^ Baltimore, Maryland, 2000-2002

**Cardiovascular Disease Risk Factors (Criteria)**	Girls Without MS, % Above Criterion	Girls With MS, % Above Criterion
Triglyceride level (≥1.1 mmol/L)	3	35
HDL cholesterol level (<1.3 mmol/L)	32	91
Fasting blood glucose level (≥6.1 mmol/L)	0	9
WC measurement (>75th percentile for age and sex)	32	94
SBP (>90th percentile for age, sex, and height)	7	41
DBP (>90th percentile for age, sex, and height)	21	82

Abbreviations: MS, metabolic syndrome; HDL cholesterol, high-density lipoprotein cholesterol; WC, waist circumference; SBP, systolic blood pressure; DBP, diastolic blood pressure.

a MS definition established by De Ferranti et al (12).

**Table 5:** Participant (N = 185) Distribution of Metabolic Syndrome Using Body Mass Index and Waist Circumference Cutpoints^a^, Baltimore, Maryland, 2000-2002

Cutpoint			No. of Participants (%)	Likelihood of Having MS	

				AOR^b^ (95% CI)	*P* Value^c^
**BMI^d^**	Exceeds (n = 118)	No MS	88 (74.6)	10.2 (2.30-45.46)^e^	.002
		MS	30 (25.4)		
	Does not exceed (n = 67)	No MS	64 (95.5)	Ref	
		MS	3 (4.5)		
**Waist circumference^f^**	Exceeds (n = 104)	No MS	75 (72.1)	24.3 (3.23-182.89)^g^	.002
		MS	29 (27.9)		
	Does not exceed (n = 81)	No MS	80 (98.8)	Ref	
		MS	1 (1.2)		

Abbreviations: MS, metabolic syndrome; AOR, adjusted odds ratio; BMI, body mass index.

a Cutpoints established by Katzmarzyk et al (8).

b Adjusted for age and ethnicity.

c *P* values derived using logistic regression.

d Distribution of girls (who exceeded and did not exceed the BMI cutpoint) with metabolic syndrome calculated using χ^2^ test, *P* <.001.

e Participants with a BMI that exceeded the cutpoint were 91% as likely as participants with a BMI that did not exceed the cutpoint to have MS.

f Distribution of girls (who exceeded and did not exceed the waist circumference cutpoint) with MS calculated using χ^2^ test, *P* <.001.

g Participants with a waist circumference that exceeded the cutpoint were 96% as likely as participants with a waist circumference that did not exceed the cutpoint to have MS.
